# Turkish version of the renal inpatient nutrition screening tool: validity and reliability for haemodialysis patients

**DOI:** 10.1017/S0007114524003192

**Published:** 2025-01-28

**Authors:** Nursena Ersoy Söke, Emine Karademir, Ebru Bayrak, Muslu Kazım Körez, Hülya Yardımcı

**Affiliations:** 1 Ankara University Institute of Health Sciences, Dışkapı Campus Şehit Ömer Halisdemir Boulevard 06110 Dışkapı Ankara, Ankara, Turkey; 2 Niğde Ömer Halisdemir University, Faculty of Health Sciences, Department of Nutrition and Dietetics, Bor/Nigde, Turkey; 3 Selçuk University, Faculty of Health Sciences, Department of Nutrition and Dietetics, Alaeddin Keykubat Campus 299/1, 42250 Selçuklu/Konya, Turkey; 4 Selçuk University, Faculty of Medicine, Department of Biostatistic, Alaeddin Keykubat Campus, 42131 Selçuklu/Konya, Turkey; 5 Ankara University, Faculty of Health Sciences, Department of Nutrition and Dietetics, Fatih Street, Tepebaşı District, No:197/A, 06300 Kecioren-Ankara, Turkey

**Keywords:** Chronic kidney disease, Haemodialysis, Malnutrition, Renal, Nutritional Assessment

## Abstract

The aim of this study was to analyse the validity and reliability of the Turkish version of the renal inpatient nutrition screening tool (Renal iNUT) for haemodialysis patients. The Renal iNUT and the malnutrition universal screening tool (MUST) were used in adult haemodialysis patients at two different centres to identify malnutrition. The subjective global assessment (SGA), regarded as the gold standard for nutritional status assessment, was utilised for comparison. Structural validity was assessed using biochemical values and anthropometric measurements, while reliability was assessed using repeated the Renal iNUT assessment. Of the 260 patients admitted, 42·3 % were malnourished (SGA score was B or C). According to the Renal iNUT, 59·6 % of the patients were at increased risk for malnutrition (score ≥ 1) and 3·8 % required referral to a dietitian (score ≥ 2). According to the MUST, 13·1 % of the patients were at increased risk for malnutrition and 8·5 % required referral to a dietitian. The Renal iNUT was found to be more sensitive in detecting increased risk of malnutrition in haemodialysis patients compared with the MUST (59·6 % *v*. 13·1 %). According to the SGA, the sensitivity of the Renal iNUT is higher compared to the MUST (89 % and 45 %, respectively). Kappa-assessed reliability of the Renal iNUT was 0·48 (95 % CI, 0·58, 0·9) and a moderate concordance was observed. The Renal iNUT is a valid and reliable nutritional screening tool for evaluating haemodialysis patients to determine their nutritional status. The use of the Renal iNUT by dietitians will contribute to the identification of malnutrition and its treatment.

Chronic kidney disease is a disease characterised by the chronic and progressive impairment of renal functions, resulting from damage and dysfunction of the kidney’s haemofiltration processes^(1)^. This disease, which affects approximately 10 % of the global population and one in two individuals over the age of 75, represents a significant global public health concern^(2)^. It was reported that the median prevalence of chronic kidney disease is 9·5 %^(3)^. In Turkey, the prevalence is 15·7 %^(4)^.

The nature of chronic kidney disease results in malnutrition in haemodialysis patients due to a combination of factors, including dialysis-related issues, psychosocial influences and a decline in physical capacity^(5,6)^. As a consequence of diminished appetite resulting from inflammatory processes and alterations in taste perception associated with uremia, these patients exhibit a deficiency in energy and protein intake^(7)^. This condition, which is associated with hypercatabolism, leads to muscle wasting and malnutrition^(8)^. Given the association between malnutrition in these patients and poor prognosis and quality of life, it is imperative to prioritise the prevention and early diagnosis of malnutrition is important^(7)^.

It is recommended that the nutritional status of haemodialysis patients be reviewed during the initial 90-day period following the commencement of dialysis and subsequently on an annual basis, or at a frequency determined by the results of a nutritional screening tool. A comprehensive nutritional assessment should include an evaluation of appetite, dietary history, biochemical parameters, anthropometric measurements and any physical symptoms that may be related to nutritional status. The use of screening and malnutrition assessment tools is strongly recommended for this purpose. Nevertheless, there are notable distinctions between nutritional screening and assessment tools. Nutritional screening tools are designed to identify the risk of malnutrition in a given population, whereas nutritional assessment tools are used to conduct a comprehensive examination of the nutritional status of individuals who are at risk of malnutrition^(9)^. Accordingly, when selecting a tool for the assessment of malnutrition, it is essential to exercise caution in interpreting the results, regardless of whether the tool is intended for screening or assessment purposes. The National Kidney Foundation recommends the use of the 7-item Subjective Global Assessment (SGA) tool for dialysis patients. The SGA is a valid and reliable tool for dialysis patients, as it addresses a range of pertinent health issues, including weight loss, food intake, gastrointestinal problems, muscle loss, loss of fat stores, oedema and functional capacity over the previous 6 months^(10,11)^. In Turkey, the 7-P SGA is also a valid and reliable tool for use with haemodialysis patients^(12)^.

The Malnutrition Universal Screening Tool (MUST) is another commonly used screening tool for malnutrition^(13,14)^. The MUST includes an assessment of BMI, body weight loss and any acute illness associated with fasting for more than 5 days. It should be noted that the sensitivity is lower in all renal inpatients, not only those undergoing haemodialysis, than it is in patients assessed using the SGA as a reference tool^(15)^. In Turkey, the MUST has also been shown to be a specific screening tool for this population, but with less sensitivity^(16)^. Given the challenges associated with utilising the SGA and the reduced sensitivity of the MUST, the renal inpatient nutrition screening tool (Renal iNUT) screening tool for malnutrition was devised with the objective of enhancing simplicity and practicality by employing a series of questions pertaining to body weight loss, BMI, appetite, food intake and nutritional support^(17)^ This study aimed to examine the validity of this nutritional screening tool, which has been demonstrated to be a valid and reliable instrument for use in renal specialist wards in the UK, in assessing the nutritional status of patients receiving haemodialysis in dialysis centres, which are common in Turkey.

## Method

**Figure 1.**
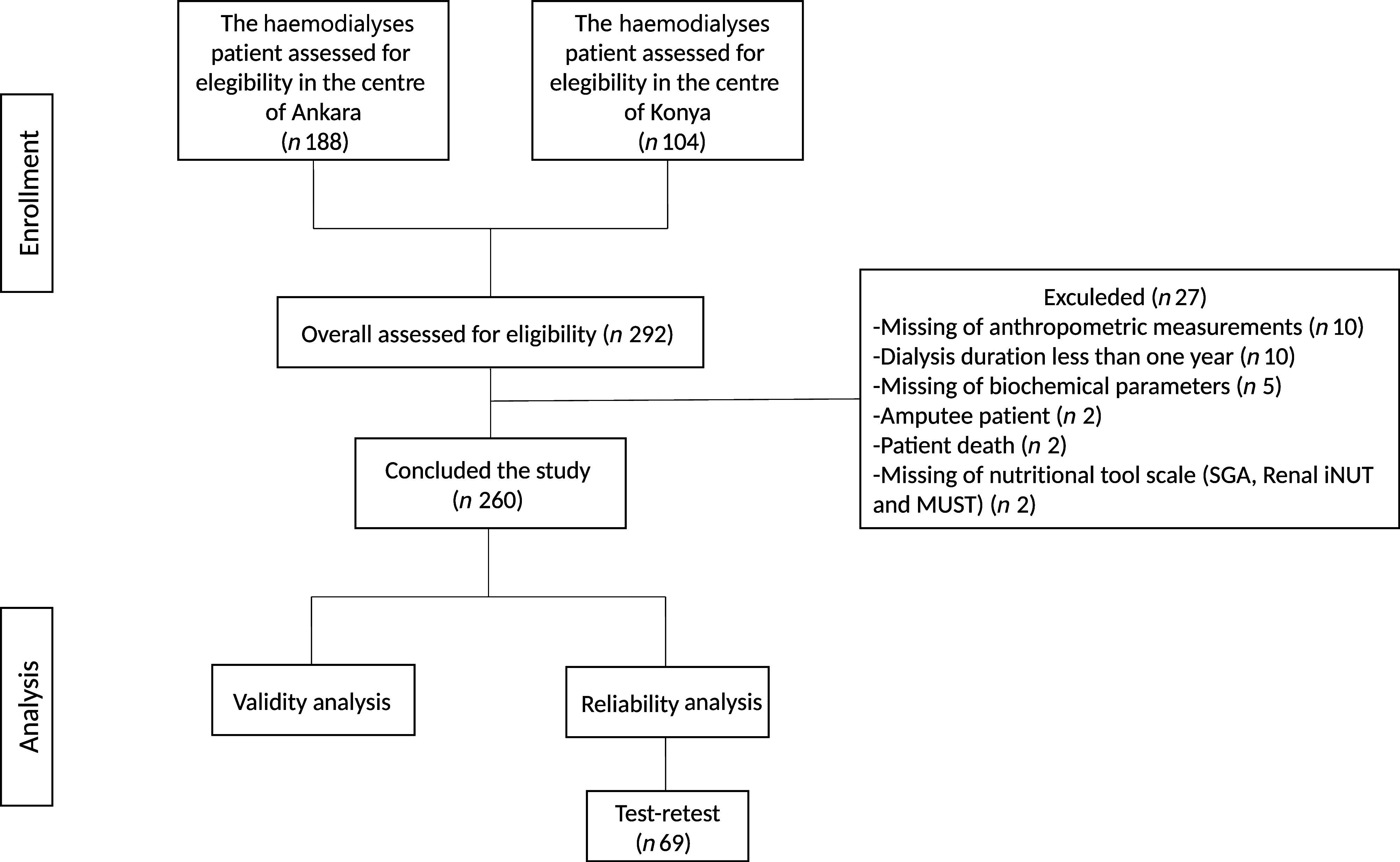
Flow diagram of the conduct of the study. Renal iNUT, Renal Inpatient Nutrition Screening Tool. Renal iNUT, renal inpatient nutrition screening tool.

The data were gathered from two private haemodialysis centres in two distinct cities within the CCC region between February and April 2022. A total of 260 volunteer patients, aged 20–65 years who had been undergoing regular haemodialysis treatment for a minimum of one year and could speak Turkish were enrolled in the study. Patients were excluded from the study if they were younger than 19 years of age, older than 65, pregnant, had hearing difficulties, an intellectual disability or were experiencing fatigue during haemodialysis (Fig. 1). The study was conducted in accordance with the ethical standards of the Ethics Board for Noninvasive Research of … University (approval no. 2022/60) and in compliance with the STROBE checklist for observational research. Prior to their participation, all subjects provided informed consent.

Permission to translate the Renal iNUT was granted via e-mail by Helena S. Jackson. A standard translation and back translation were conducted. In the preliminary translation phase, two Turkish-speaking translators proficient in English translated the screening tool independently into Turkish. The discrepancies between the two versions were collectively addressed by the research team. Another bilingual translator, who was not familiar with the English version, translated the screening tool back into English. A total of ten experts in the field of nutrition were invited to participate in a content validity test of Renal iNUT. The experts were provided with the expert form via email and asked to rate the simplicity, clarity, appropriateness and necessity of each question. In light of the expert opinions, the content validity index was calculated to be 0·76. This value indicates that the scale is deemed to possess content validity^(18)^. The comments provided by the experts were evaluated by the research teams. Following this evaluation, a pilot study was conducted on twenty individuals without making any significant alterations to the screening tool. Once the necessary corrections had been made, the final version of the screening tool was produced.

The patients were queried regarding their age, sex, date of diagnosis of chronic kidney disease, duration of dialysis treatment and the presence of any chronic diseases. The Charlson comorbidity index was calculated based on the patients’ medical histories^(19)^. The biochemical parameters were used in the standard clinical practice. Additionally, an expert measured the following variables after dialysis: dry body weight, height, mid-upper arm circumference (MUAC), handgrip strength (HGS) and triceps skinfold thickness. Patients were weighed without shoes or outer garments in order to determine their dry body weight. This measurement was taken with the use of an accurate Seca 799 scale, calibrated in 0·1-kilogram increments^(20)^. The patients’ heights were measured with their feet bare and in an upright position, with their heads aligned with the Frankfurt plane, using a portable stadiometer (Seca 769)^(21)^. The MUAC was measured by having the subjects adopt an upright stance and flex the arm at the elbow to achieve a 90° angle. The midpoint between the acromion and the olecranon process was marked, and the mean humeral circumference was measured with a tape measure^(22)^. In the triceps skinfold thickness, the subjects were instructed to flex their elbows at a 90° angle, parallel to the floor, in an upright position. The right MUAC was determined and marked. Subcutaneous adipose tissue was then held with the thumb and index finger at the centre of the right mid-dorsum of the upper arm and measured with a Holtain brand caliper^(23)^. The HGS was measured on at least three occasions from the arm devoid of fistula with the use of a Takei digital dynamometer. The procedure was conducted with the shoulder in a state of adduction, with the elbow maintained at a flexion angle of 90°. The highest value obtained was deemed to be the patient’s HGS^(24)^.

The Renal iNUT is a renal-specific nutrition screening tool comprising five questions developed by Jackson *et al.* in 2019. The total score obtained from the Renal iNUT is indicative of the necessity for further evaluation and intervention. A score of 0 indicates that weekly screening is sufficient, while a score of 1 suggests the need for follow-up of high-risk patients. A score of ≥ 2 indicates that the patient should be referred to a dietitian. In this screening tool, a score of ≥ 1 was identified as indicative of an increased risk of malnutrition, consistent with the findings of the original study^(17)^. A total of 6–7 points from SGA 7P indicates that the patient is well nourished (A), 3–5 points indicate moderate malnutrition (B) and 1–2 points indicate severe malnutrition (C)^(11)^. In this study, SGA A was classified as indicating a low nutritional risk, SGA B as indicating an increased nutritional risk and SGA C as indicating the need for referral to a dietitian. Furthermore, SGA B + C was classified as indicating an increased risk of malnutrition. The MUST consists of three questions. A total score of 0 is indicative of low risk, 1 is suggestive of increased nutritional risk and a score of ≥ 2 is indicative of the necessity for referral to a dietitian^(25)^. For MUST and Renal iNUT, a score of 1 or greater was classified as indicative of an increased risk for malnutrition. All screening and assessment tools were applied by nurses and dietitians in the centres and by experts on the study team.

All statistical analyses were performed with the R software, version 4.2.1. (The *R* Foundation for Statistical Computing, Vienna, Austria; https://www.r-project.org). The Shapiro–Wilk normality test, and Q-Q plots were used to evaluate the normality of the data. In addition, Levene’s test was used to check the homogeneity of variances. Numerical variables are expressed as mean (standard deviation) or median with interquartile range (25th percentile–75th percentile), as appropriate. One-way ANOVA (analysis of variances) and the Kruskal–Wallis tests were performed to determine whether there was a statistically significant difference between the Renal iNUT categories, and these statistical test results were also used to examine the construct validity of the Renal iNUT. Subsequently, *post hoc* comparisons were conducted using the Tukey HSD and Bonferroni-corrected Dunn tests, respectively, in cases where the variables were found to be significant following the aforementioned tests. To ascertain the diagnostic efficacy of the Renal iNUT method for the identification of malnutrition as determined by the SGA method, receiver operating characteristics curve analysis and statistical diagnostic measures (sensitivity, specificity, positive predictive value, negative predictive value, diagnostic accuracy and kappa value) were employed. Furthermore, we conducted a comparative analysis of the diagnostic efficacy of the Renal iNUT method and the MUST method for the identification of malnutrition risk in haemodialysis patients. The McNemar test was performed to compare the sensitivity and specificity of the Renal iNUT and MUST methods, and the weighted generalised score test was also applied to compare the negative and positive predictive values of these methods. We examined the interrater reliability of the Renal iNUT method using weighted kappa value. A *P*-value of <5 % was considered statistically significant.

## Results

A total of 260 patients (52 % male and 48 % female) who received haemodialysis at two private dialysis centres in Ankara (*n* 167, 64·2 %) and Konya (*n* 93, 35·8 %) were included in the study. The mean age of the patients was 62·91 (13·39) years. The data regarding anthropometric measures and biochemical parameters are presented in Table 1.

**Table 1.** Various biochemical parameters and anthropometric measurements of haemodialysis patients

Variables	Median	IQR
Body weight (post dialysis)	69·0	59·0–69·5
Height (m)	1·65	1·59–1·75
BMI (kg/m^2^)	25·5	21·9–28·9
TST (mm) (*n* 260)	11·0	7·4–17·0
TST, women (mm) (*n* 125)	17·0	12·0–20·0
TST, men (mm) (*n* 135)	8·4	6·2–11·0
HGS (kg) (*n* 260)	16·1	10·0–23·2
HGS, women (*n* 125)	10·9	7·5–15·3
HGS, men (kg) (*n* 135)	22·3	16·5–27·9
Serum albumin (g/l)	3·7	3·6–3·9
Total protein (g/l)	6·8	6·5–7·1
C-reactive protein (mg/dl)	6·6	2·7–12·7
Creatinine (mg/dl)	2·6	2·0–3·3
Potassium (mEq/l)	3·5	3·2–3·8
Phosphor (mg/dl)	4·8	4·0–5·5
Ca (mg/dl)	8·6	8·2–9·0
Total cholesterol (mg/dl)	163·0	146·0–187·0
TAG (mg/dl)	136·0	94·0–185·0
HDL (mg/dl)	35·5	31·0–43·3
LDL (mg/dl)	91·0	68·6–114·0
Charlson comorbidity index	5·0	3·0–6·0

TST, triceps skinfold thickness; HGS, handgrip strength.

**Figure 2.**
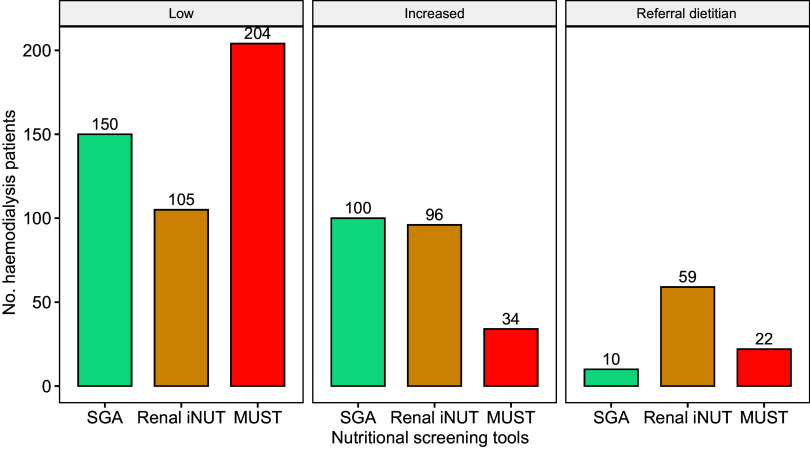
Subjective global assessment (SGA), renal inpatient nutrition screening tool (Renal iNUT) and malnutrition universal screening universal (MUST) for patients with low nutritional risk, increased nutritional risk and referral to a dietitian.

According to SGA category A, screening tool Renal iNUT = 0 and MUST = 0, 57·7 %, 40·4 % and 78·5 % of haemodialysis patients have low nutritional risk, respectively. According to SGA B, Renal iNUT = 1 and MUST = 1, respectively, 38·5 %, 36·9 % and 13·1 % of the patients who increased nutritional risk and according to SGA category C, Renal iNUT ≥ 2 and MUST ≥ 2 scores, it was found that 3·8 %, 22·7 % and 8·5 % of the patients needed to be referred to a dietitian. In addition, the number of patients found to be at increased risk for malnutrition according to Renal iNUT (*n* 96) was more than double the number of patients with MUST (*n* 34) (Fig. 2).

A comparison of Renal iNUT and MUST in patients with low and severe malnutrition (SGA B + C) (42·3 %) reveals a higher prevalence of increased nutritional risk with Renal iNUT ≥ 1 (37·7 %) than with MUST ≥ 1 (19·2 %) (Table 2).

**Table 2.** The validity of the renal inpatient nutrition screening tool (renal iNUT) and malnutrition universal screening tool (MUST) according to clinical outcome category, following comparison with subjective global assessment in haemodialysis patient population (*n* 260)

	Renal iNUT		MUST		*P* value
	iNUT = 0 (*n* 105)	iNUT ≥ 1 (*n* 155)	MUST = 0 (*n* 204)	MUST ≥ 1 (*n* 56)	
SGA (Gold standard)					
SGA A (*n* 150)	93	57	144	6	
SGA (B + C) (*n* 110)	12	98	60	50	
ROC curve analysis					
AUC (95 % CI)	0·755	0·696, 0·815	0·707	0·640, 0·744	
* P* value	< 0·001		< 0·001		
Youden index (*J*)	0·511		0·415		
Statistical Diagnostic Measures					
Sensitivity (95 % CI)	89	82, 94	45	36, 55	< 0·001^*^
Specificity (95 % CI)	62	54, 70	96	91, 99	< 0·001^*^
Accuracy (95 % CI)	73	68, 79	75	69, 80	
PPV (95 % CI)	63	55, 71	89	78, 96	< 0·001^†^
NPV (95 % CI)	89	81, 94	71	64, 77	< 0·001^†^
* κ*	0·484		0·444		

AUC, area under the curve; PPV, positive predictive value; NPV, negative predictive value; *κ*, kappa value.

* McNemar test.

† Weighted generalised score test.

**Figure 3.**
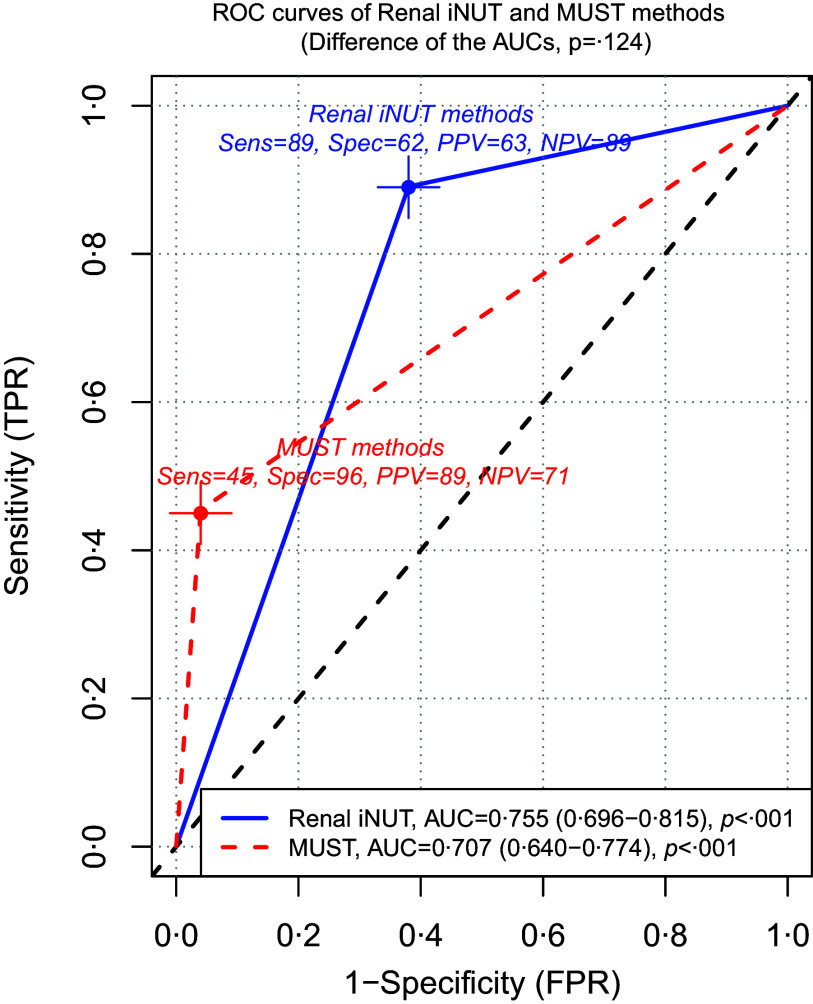
The receiver operating characteristics (ROC) curves for the comparison of Renal iNUT and MUST methods in diagnosising of malnutrition, which is assessed using the SGA method. The reference line was colored with black; the ROC curve of Renal iNUT was colored with blue; ROC curve of MUST was colored with blue. DeLong’s test revealed that there was no statistically significant difference between AUC for methods of malnutrition screening tools (*P* = 0·124). MUST, malnutrition universal screening universal; Renal iNUT, renal inpatient nutrition screening tool; ROC, receiver operating characteristics.

A comparison of Renal iNUT and MUST sensitivity, as reported by SGA, revealed that Renal iNUT sensitivity was statistically significantly higher than that of MUST (89 % *v*. 45 %, *P*-value < 0·001). The Kappa coefficient of concordance, which indicates the degree of concordance between SGA and Renal iNUT, was calculated to be 0·484, indicating a moderate level of concordance (Table 2). Figure 3 illustrates the results of the receiver operating characteristics curve analysis, which indicates that Renal iNUT and MUST are effective in predicting risk malnutrition in haemodialysis patients (AUC = 0·755 (95 % CI, 0·696, 0·815) and AUC = 0·707 (95 % CI, 0·640, 0·774), respectively).

**Figure 4.**
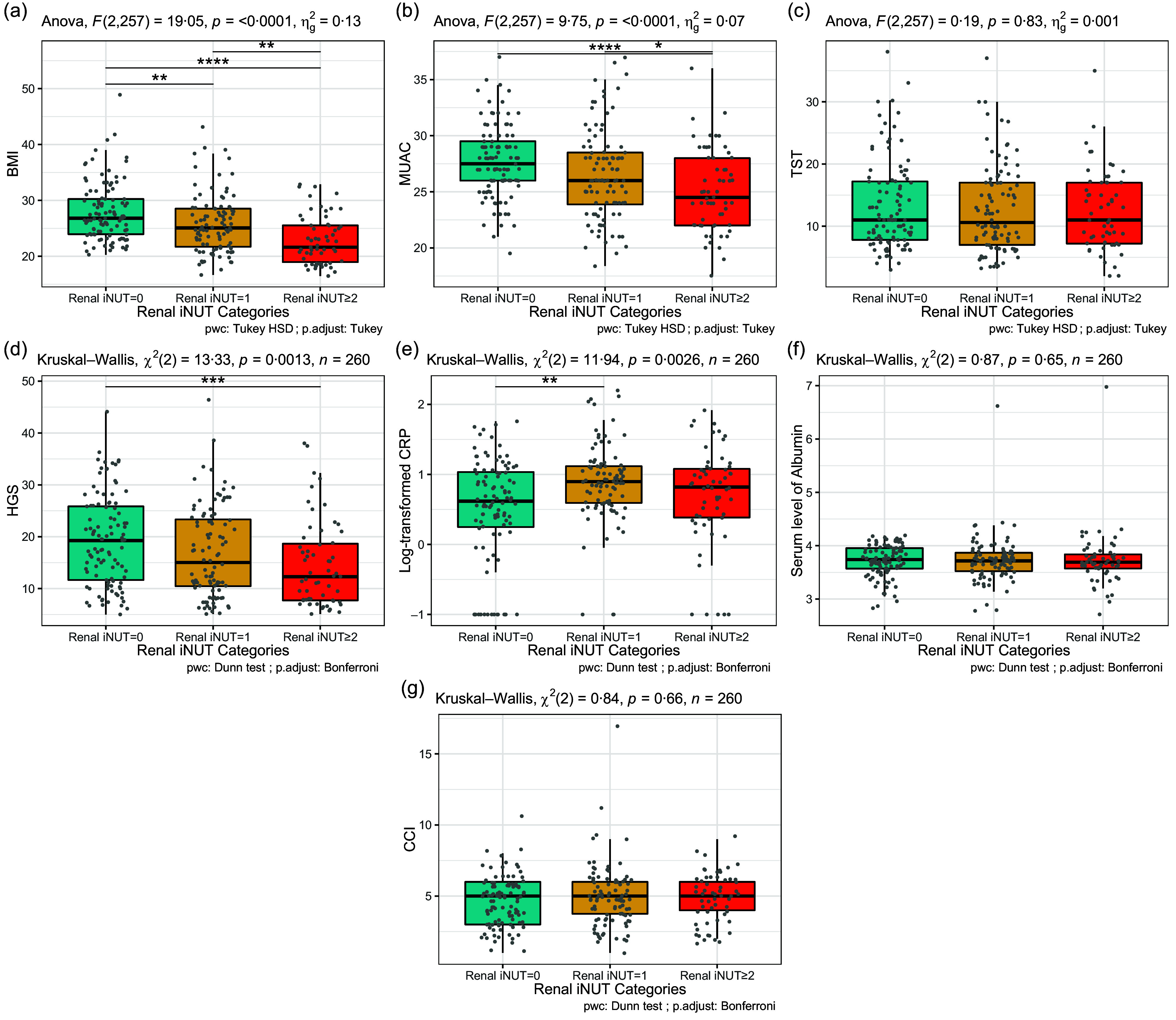
The box plot of anthropometrical measurements and biochemical parameters in patients with haemodialysis. Horizontal lines in each box indicate the median. Data were represented as median with interquartile ranges. The one-way ANOVA followed by Tukey HSD, and Kruskal–Wallis *H* test followed by Dunn *post hoc* test with Bonferroni correction was used for comparisons between Renal iNUT categories. CCI, Charlson comorbidity index; CRP, C-reactive protein; HGS, handgrip strength; MUAC, middle upper arm circumference; Renal iNUT, receiver operating characteristics; TST, triceps skinfold thickness.

To evaluate the construct validity of Renal iNUT, anthropometric measurements and biochemical parameters were evaluated in accordance with the patients’ Renal iNUT scores. As the Renal iNUT score increased, there was a statistically significant decrease in BMI in each Renal iNUT category (Fig. 4(a)). In the group referred to a dietitian (Renal iNUT score ≥ 2), there was a statistically significant decrease in MUAC (Fig. 4(b)). In addition, the handgrip scores of individuals at low nutritional risk were statistically significantly higher than those who were referred to a dietitian (Fig. 4(d)). In the biochemical parameters, a significant reduction in C-reactive protein levels was observed in subjects with Renal iNUT = 0 compared with those with Renal iNUT = 1 (Fig. 4(e)) (Table 3). The weighted kappa for the repeated Renal iNUT assessment in a subsample of sixty-nine subjects was 0·608 (95 % CI, 0·449, 0·766) for scores 0–3, indicating good and substantial agreement between the scores.

**Table 3.** Differences between Renal iNUT scores of anthropometric measurements and parameters (*n* 260)

	Renal iNUT categories						*P*-value
	Renal iNUT = 0 (*n* 105)		Renal iNUT = 1 (*n* 96)		Renal iNUT ≥ 2 (*n* 59)		
	Mean	sd	Mean	sd	Mean	sd	
Anthropometric measures							
BMI	27·94	5·20^a^	25·81	5·23^b^	22·90	4·32^c^	< 0·001^*^
MUAC	27·70	3·25^a^	26·52	4·06^a^	25·08	3·66^b^	< 0·001^*^
TST	13·12	7·18	12·70	7·04	12·45	6·41	0·827^*^
	Median	IQR	Median	IQR	Median	IQR	
HGS	19·25	11·65–5·85^a^	15·02	10·45–23·32	12·30	7·72–18·65^b^	0·001^†^
Biochemical parameters							
CRP	4·15	1·7–10·82^a^	7·90	3·92–13·10^b^	6·60	2·42–12	0·003^†^
Serum albumin	3·74	3·57–3·96	3·71	3·52–3·87	3·69	3·57–3·84	0·648^†^
CCI	5	3–6	5	3·75–6	5	4–6	0·657^†^

Renal iNUT, renal inpatient nutrition screening tool; MUAC, middle upper arm circumference; TST, triceps skinfold thickness; HGS, handgrip strength; CRP, C-reactive protein, CCI, Charlson comorbidity index.

Data were expressed as mean (standard deviation) or median (interquartile range).

Different small superscripts in each row indicate that statistically significant difference between groups after multiple comparison.

* One-way ANOVA followed by Tukey HSD test.

† Kruskal–Wallis followed by Dunn test with Bonferroni correction.

## Discussion

In this study, the Renal iNUT screening tool, developed by Jackson *et al.* (2019), was adapted for the purpose of determining the prevalence of malnutrition among Turkish haemodialysis patients. The tool was applied to patients receiving treatment in haemodialysis centres. Anthropometric measurements and biochemical parameters were analysed, and the Renal iNUT was found to be a valid tool for this purpose. Furthermore, this tool has been demonstrated to be reliable for the Turkish population when used for repeated Renal iNUT assessments. Additionally, the Renal iNUT has been shown to be more sensitive for this population than the MUST, a commonly used screening tool.

It is crucial to evaluate the nutritional status of patients undergoing dialysis for chronic kidney disease and to identify instances of malnutrition through the assessment of multiple parameters^(25)^. Nutritional screening tools facilitate the assessment of nutritional status and the evaluation of the efficacy of implemented interventions^(26)^. Malnutrition may result from deficiencies, excesses and imbalances in energy and nutrient intake in these patients. Such imbalances can lead to alterations in body composition (e.g. changes in fat and muscle percentage), a decline in physical and mental functions and complications^(27)^. It is, therefore, of great importance to be able to identify cases of malnutrition at an early stage and to take appropriate action.

The SGA is a valid and reliable nutritional assessment tool used in haemodialysis patients^(28)^. In our study, the prevalence of malnutrition risk was 42·3 % among haemodialysis patients according to the SGA. A recent meta-analysis revealed that the prevalence of malnutrition among haemodialysis patients according to the SGA ranges between 28 % and 54 %^(29)^. In this sample, the percentage in Turkey was reported to be 29·3^(30)^. According to the Renal iNUT, which is a new screening tool for renal patients, 45·4 % of haemodialysis patients are at malnutrition risk^(31)^, whereas in our study, this ratio was found to be 59·6 %. The Renal iNUT questionnaire encompasses inquiries pertaining to BMI, weight loss, dietary intake and the utilisation of nutritional supplements. In addition to other components, the assessment of supplement use, particularly in renal patients, may be crucial for identifying the risk of malnutrition. According to the MUST, which is another screening tool used in this study, 21·6 % of the patients were at risk of malnutrition. The sensitivities of the Renal iNUT and MUST were reported to be 89 % and 45 %, respectively, compared with the SGA gold standards. The Renal iNUT has been demonstrated to be a statistically significantly better predictor of malnutrition in haemodialysis patients than the MUST. In a study conducted by Jackson *et al.* (2019), Renal iNUT was found to have a sensitivity of 92·1 %, while the sensitivity of MUST was determined to be 44·4 %. The findings of both the original study and our own investigation indicate that the Renal iNUT is a more effective screening instrument than the MUST in patients undergoing haemodialysis^(17)^.

Anthropometric measures can be incorporated into a comprehensive nutritional assessment in people with kidney disease^(10)^. In patients undergoing haemodialysis, oedema frequently arises for a number of reasons, including low albumin levels, excessive fluid intake, diminished cardiac function and hypotension^(32)^. The presence of oedema can impede the accurate assessment of malnutrition by obscuring body weight. To mitigate this potential confounding factor, this study employed a methodology wherein anthropometric measurements were obtained following the completion of haemodialysis. As per the Renal iNUT categories, the BMI was 27·94 kg/m^2^ (Renal iNUT = 0), 25·81 kg/m² (Renal iNUT = 1) and 22·90 kg/m² (Renal iNUT ≥ 2), with a notable decline in BMI as the risk of malnutrition increased. Similarly, in another study, a negative correlation was observed between BMI and the score on the MUST, with a score of 0–1 indicating a BMI of 23·7 kg/m² and a score of > 2 indicating a BMI of 17·3 kg/m²^(16)^. In patients undergoing haemodialysis, both underweight and obesity are associated with an elevated risk of mortality, as indicated by BMI^(10)^. Accordingly, the objective is to ascertain the precise body weight of haemodialysis patients and ensure that their BMI values remain within the normal range.

In addition to BMI, body composition, which is composed of extracellular fluid, muscle mass and fat mass, is a significant factor in the assessment of nutritional status. In order to evaluate body fat composition, it is recommended that renal patients undergo skinfold thickness measurement, as this method is both simple and cost effective^(10)^. The present study did not identify a statistically significant correlation between the Renal iNUT classification and the triceps skinfold thickness results^(33)^. Handgrip strength, which is a sign of adequate energy and protein intake, is also indicative of skeletal muscle function and nutrition. In this instance, the recommended approach is to utilise the HGS method, which is a straightforward, non-invasive and highly effective technique for evaluating muscle function^(34,35)^. In our study, the mean HGS value was found to be 22·3 kg (range: 16·5–27·9 kg) in males and 10·9 kg (range: 7·5–15·3 kg) in females. While the mean HGS score in various Turkish studies is higher than that observed in the present study, the HGS score of males is comparable to that of females^(36,37)^. As reported by Renal iNUT, the HGS score of the cohort who had consulted with a dietician was found to be statistically significantly lower in comparison to the cohort that was deemed to be well-nourished. These findings reinforce the notion that despite patients exhibiting normal BMI values, muscle mass should be assessed independently, as BMI is not a reliable indicator of body composition^(38)^. MUAC, which is also an indicator of body fat, varies between Renal iNUT categories. As a result, both BMI and HGS along with MUAC values differ among Renal iNUT classifications. This shows that as the risk of malnutrition increases, HGS and MUAC levels decrease in parallel with BMI.

In patients with renal disease, biomarkers such as serum albumin are also useful additional methods for assessing nutritional status^(10)^. Although albumin levels did not demonstrate significant discrepancies between categories of nutritional risk as defined by Renal iNUT, elevated C-reactive protein levels were observed in individuals at elevated nutritional risk. Moreover, there was no correlation between albumin levels and nutritional status, even when C-reactive protein levels were elevated. This may provide a novel perspective on the use of C-reactive species in lieu of albumin in SGA or other global measures of nutritional status. The occurrence of inflammatory markers in these patients can be attributed to a number of factors, including infection of the fistula, the composition of the dialysate, filtration processes, chronic infection and inadequate nutritional intake^(39)^. Despite the absence of studies demonstrating the validity and reliability of inflammatory markers as a standalone assessment of nutritional status, there is evidence indicating a correlation between inflammatory markers and other nutritional markers^(40)^. However, because these markers are also influenced by factors other than diet, biomarkers should be assessed together rather than separately to evaluate nutritional status.

The principal strength of this study is that it was conducted at two different centres in Turkey, and that a number of anthropometric measurements and routine biochemical parameters were evaluated. Furthermore, the repeat test conducted 5 days later served to corroborate the reliability of Renal iNUT. The researchers, who are dietitians, applied the Renal iNUT. A limitation of the study is that the content and ease of use of the screening tool were not evaluated by the nurses or the dietitian at the dialysis centre. Furthermore, it should be noted that the present study, which was conducted at two haemodialysis centres, may yield different results when compared with those observed in inpatients. It is possible that alterations in the environment may result in a shift in the characteristics of the patient population. The population under investigation comprises patients who reside in their own homes and who visit the centre for dialysis sessions. It would be beneficial to conduct further studies with larger samples, including inpatients from diverse regions within Turkey.

The Renal iNUT nutritional screening tool is a valid and reliable method for assessing the nutritional status of patients undergoing haemodialysis. Based on the results, it was recommended that iNUT be included in usual care by nurses or dietitians. The use of Renal iNUT allows the screening of nutritional status and allows the early treatment of malnutrition once it has been identified as a risk. The Renal iNUT is a specific screening tool developed for patients with renal diseases. It is recommended that future studies include a greater variety of samples, comprising renal patients receiving different medical treatments such as peritoneal dialysis.
